# Sex biased expression and co-expression networks in development, using the hymenopteran *Nasonia vitripennis*

**DOI:** 10.1371/journal.pgen.1008518

**Published:** 2020-01-27

**Authors:** Alfredo Rago, John H. Werren, John K. Colbourne

**Affiliations:** 1 School of Biosciences, The University of Birmingham, Birmingham, United Kingdom; 2 Department of Biology, University of Rochester, Rochester, NY, United States of America; New York University, UNITED STATES

## Abstract

Sexual dimorphism requires regulation of gene expression in developing organisms. These developmental differences are caused by differential expression of genes and isoforms. The effect of expressing a gene is also influenced by which other genes are simultaneously expressed (functional interactions). However, few studies have described how these processes change across development. We compare the dynamics of differential expression, isoform switching and functional interactions in the sexual development of the model parasitoid wasp *Nasonia vitripennis*, a system that permits genome wide analysis of sex bias from early embryos to adults. We find relatively little sex-bias in embryos and larvae at the gene level, but several sub-networks show sex-biased functional interactions in early developmental stages. These networks provide new candidates for hymenopteran sex determination, including histone modification. In contrast, sex-bias in pupae and adults is driven by the differential expression of genes. We observe sex-biased isoform switching consistently across development, but mostly in genes that are already differentially expressed. Finally, we discover that sex-biased networks are enriched by genes specific to the *Nasonia* clade, and that those genes possess the topological properties of key regulators. These findings suggest that regulators in sex-biased networks evolve more rapidly than regulators of other developmental networks.

## Author summary

How can males and females of the same species be so different, despite sharing the same genes? Several explanations for this question rely on the presence of sex-specific chromosomes (XY or ZW), but this is not always the case. Wasps lack sex chromosomes; individuals that carry a single copy of the genome develop into males, while individuals that carry two copies develop into females. Since male and female wasps have the same set of genes, differences between the sexes must be caused by different uses of the same genes (bias) between sexes. We thus choose to study how sex-biased gene networks work and evolve in wasps. We detect bias across several gene-gene interactions at the earliest stages of development, when few individual genes are biased. This suggests that bias in the balance between genes, rather than bias in individual genes, can trigger developmental differences between sexes. We also find that potential regulatory genes in sex-biased networks evolve faster than regulators in networks that are shared between males and females. Together, our findings suggest that re-wiring interactions in gene networks play a key role in the functioning and evolution of differences between sexes.

## Introduction

Sexual differentiation during development involves many genes that are shared between sexes (i.e. genes on the autosomes) and therefore must be caused by different use of the same genes. Indeed, gene expression regulation and isoform switching are fundamental for sexual differentiation. For example, sexual development can be induced, even if the organism lacks the chromosomes normally associated with that sex [1,2]. Induction of a specific sexual phenotype can be achieved in the laboratory, either by changing the expression of sex-determining genes [3–6] or by administering hormones in the right developmental phase [7–9].

In nature, numerous organisms lack sex-chromosomes altogether, demonstrating that genes located exclusively on sex-specific chromosomes are not necessary for the evolution of phenotypic differences between sexes [1,10–14]. Rather, phenotypic differences between sexes seem to evolve whenever the selective pressures between the two are different (sexually antagonistic selection or conflict, see [15–18]). These observations show that the same developmental components are often involved in the formation of different sexual traits, limiting the potential to optimize either sex independently (pleiotropic constraint, or intra-locus sexual conflict). Characterizing differences in gene expression throughout the development of sexually dimorphic phenotypes is thus necessary to understand the regulatory variation that enables phenotypic diversification when selection on genes differ in males and females (i.e. there are conflicting selection pressures).

Sex-biased gene expression is by far the most studied among the mechanisms that generate differences between sexes. Most studies so far focused on measuring differences in the magnitude of gene expression only between adult males and females, and primarily in organisms with sex chromosome based sex determination [19–21]. However, the focus on adult gene expression does not reveal genes and gene networks that are sex-biased only during embryonic and pre-adult stages of development [22–24]. The detection of pre-adult sex-bias is also required to detect which genes are male-biased in some stages and female-biased in others–a scenario that can also result in pleiotropic constraint (see previous paragraph). Several studies have shown that the expression of different transcripts from the same gene can also be sex-biased [25–27], and that sex-biased isoform switching affects sex determination [6,28] and sexual development [20]. At the molecular level, isoform switching can be induced via several mechanisms, which include alternative splicing and the use of alternative transcriptional start sites (TSSs). While each of those processes involves distinct molecular mechanisms, they have comparable effects on the interaction between gene evolution and expression. Due to these reasons, we choose to study sex-biased isoform switching without discriminating between the underlying mechanisms that induce it.

The co-expression of multiple genes can result in effects that are qualitatively and quantitatively different from the sum of the effects of each gene [29–31]. For example, genes can have identical average expression levels in males and females, yet still cause a sex-specific effect if they are differentially co-expressed with other genes in one sex [32,33]. These interactions cannot be detected by independently testing transcripts, but can be identified *via* differential correlation analyses on transcriptional modules, or differential cluster correlations [34,35].

**Fig 1:**
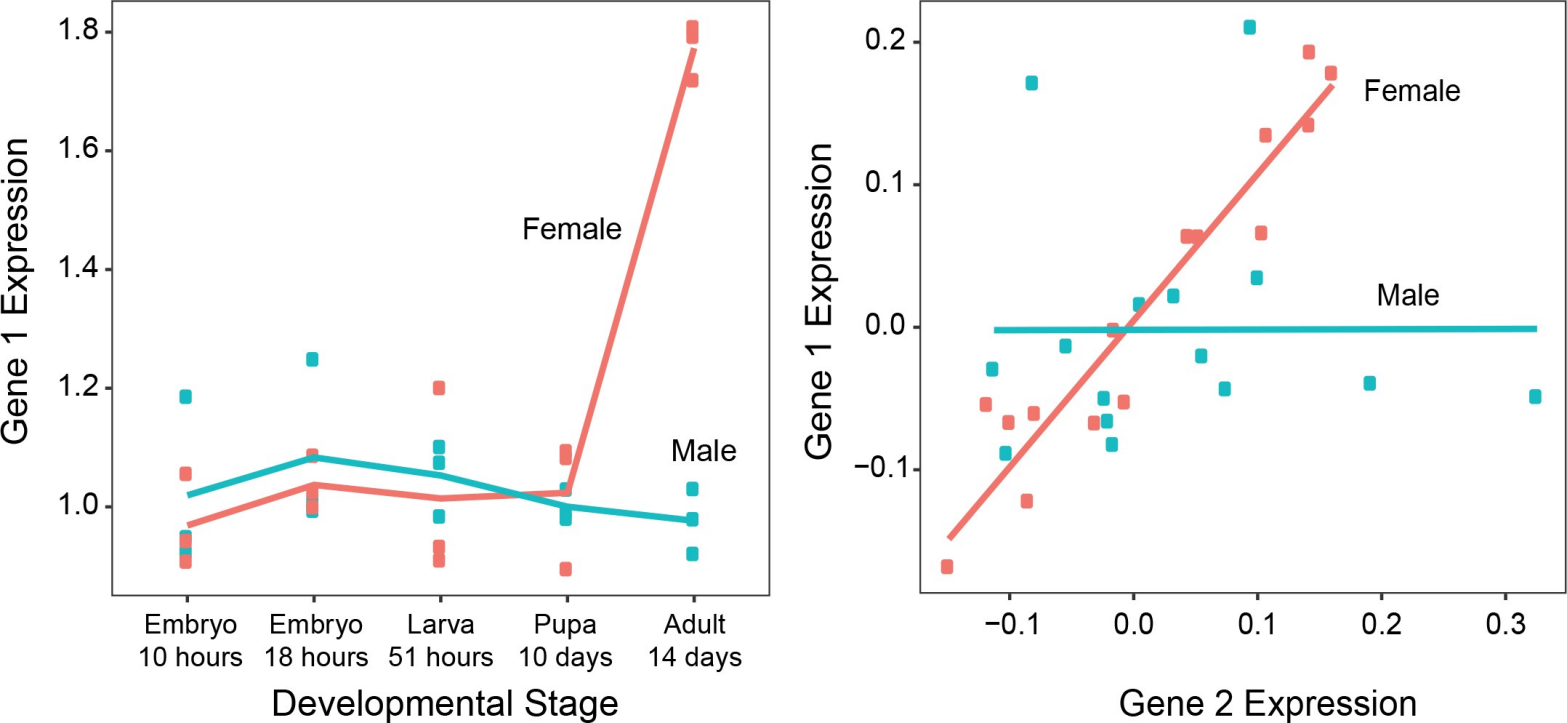
Examples of differentially expressed (left) and differentially correlated (right) clusters. Each dot represents a biological replicate of a gene’s expression value. In the left panel, the expression levels of the same gene are compared between the sexes at each stage. DE is present if the gene expression values between the sexes differ. In the right panel, the expression levels of two genes are compared across samples for all stages. DC is present if the correlation between the two genes differs between the sexes.

Differential cluster correlation (DC, right panel on Fig 1) is different from (and complimentary to) differential cluster expression (DE, left panel on Fig 1). Differential cluster expression measures the average fold expression change of a group of genes, and is thus analogous to gene-level differential expression [36]. By contrast, differential cluster correlation measures the proportion of sex-specific interactions within the cluster [37,38]. Since interactions require multiple genes, differential cluster correlation has no gene-level analog and is an emergent property of the transcriptional cluster. Due to the additional challenges involved in the study of interactions, few studies have attempted to systematically measure differential cluster correlations between sexes [33,39]. As such, while most researchers recognize the potential importance of such interactions in sex-determination and sexual development, their role is still largely unexplored.

We describe the developmental dynamics of these three forms of gene regulation (gene expression, isoform switching and co-expression) in the development of the jewel wasp *Nasonia vitripennis*. *Nasonia* has several advantages for investigating sex differential gene expression across development. First, like other hymenopterans (ants, bees, and wasps) *Nasonia* has haplodiploid sex determination [40]: males and females have the same genomes (there are no sex chromosomes) and sex differentiation is due to gene expression changes in the identical set of genes shared by the two sexes. Second, *Nasonia* can be readily inbred to produce nearly completely isogenic lines, further reducing genetic differences between individuals [41,42]. Finally, haplodiploidy also allows the collection of exclusive male and nearly exclusive female samples during the early stages of development, since (under our experimental conditions) virgin females produce only sons and mated females produce ~85–100% daughters. We can thus characterize the differences in gene expression between sexes before the onset of phenotypic differences [43].

*Nasonia* belongs to the “megadiverse”hymenopteran superfamily Chalcidoidea (henceforth referred to as “chalcids”), which is estimated to contain approximately 500,000 species [44,45]. Most chalcids are small parasitoid wasps that prey on other arthropods, and play vital roles in regulating natural and agricultural ecosystems. *Nasonia* is used extensively in genetic, developmental, and evolutionary research. *Nasonia*’s genome was the second hymenopteran to be sequenced [46] after that of the Honeybee [47]. Several other hymenopteran genomes have been sequenced since [48], enabling a study of their genomic features in a broader phylogenetic context.

We use the *Nasonia* model system to investigate genome-wide sex differences in gene expression, isoform switching, and differential co-expression of genes across development from early embryonic stage to adulthood. We provide a first genome-wide characterization of *Nasonia*’s sex-specific gene-gene transcriptional interactions via co-expression analysis across development. Finally, we examine the phylogenetic age of genes involved in these networks to investigate how they have evolved.

## Results

**Fig 2:**
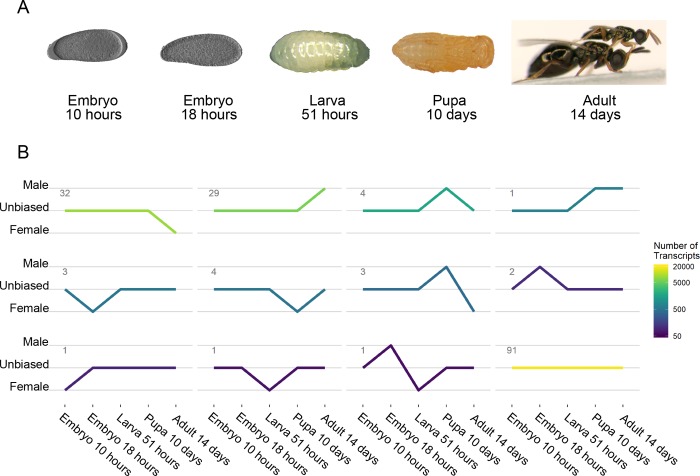
Differential expression (DE) of transcriptional clusters across developmental time from embryo (left) to adult (right). (A) Images of *Nasonia* at each stage that is sampled. Adult stage shows both male (top) and female (bottom). (B) Patterns of sex-bias in each developmental stage. Each line represents a group of clusters that shares the same pattern of significant sex-bias, tested using linear models (see Methods Section). The lines that have an upward trajectory are significantly male-bias, while lines of that have a downward trajectory are significantly female-bias. The number of clusters that share these representative expression patterns is shown at the top left of each line. The total number of transcripts included in each cluster is represented by colour (lighter for more transcripts, darker for less).

We compared the gene expression of male and female *Nasonia* across five developmental stages: early embryo, late embryo, larva, pupa and adult (see Fig 2A), using high density long oligonucleotide genome-wide tiling-path microarrays. The tiling-path microarray provide high density coverage of the non-repetitive portion of the genome, with >2 million oligonucleotides ranging in length of 50–75 and an average overlap of 30 nucleotides per adjacent oligonucleotide. Since gene expression varies greatly between different developmental stages, we perform comparisons between sexes only within each stage (see Methods). We distinguish between genes and transcripts in all our analyses; gene expression refers to the total production of RNA from a single genomic locus. Relative transcript expression instead refers to the proportion of RNAs from a single locus that contain a specific subset of exons, also known as isoform fraction or splicing ratio. This distinction allows us to study both the impact of gene regulation (via gene expression) and that of isoform switching (via relative transcript expression). Raw microarray data is available on NCBI with accession number GSE44701.

### Few loci show switches between male and female bias

Across the five developmental stages, 6041 out of 14,149 genes (43%) display sex-biased expression or isoform switching when tested individually in at least one stage (**Table 1**). Male-biased transcripts are prevalent at the pupal stage, whereas female-biased genes are prevalent at the adult stage. This pattern reflects the fact that spermatogenesis occurs primarily during the pupal stage and oogenesis occurs primarily in adults [49]. Since female gonads are much larger than males’, tissue bias is likely to have a large impact on the differential gene expression between the sexes observed in whole-body samples. We therefore specifically identified functional germline genes by measuring their differential expression in dissected ovaries and testes (Table 2 and S1 File). Overall, transcripts enriched in gonads contribute to a minor amount of the total gene expression we observe in whole organisms, with the exception of male adults and pupae.

**Table 1:** Number of sex-biased genes and transcripts at each developmental stage, compared with total number of genes or transcripts expressed at that stage. Numbers within brackets show the proportion of genes or transcripts that are male or female-biased at the given stage, compared with the total number of expressed genes or transcripts. Genes are counted as sex-biased if at least one of their transcription or splicing nodes is sex-biased. For the expression threshold used in this table, refer to the materials and methods.

Stage	Sex-biased Genes			Sex-biased Transcripts		
	Male	Female	Expressed	Male	Female	Expressed
Embryo 10 hours	26 (0.34%)	145 (1.9%)	7646	29 (0.17%)	174 (1.0%)	17,035
Embryo 18 hours	187 (2.1%)	185 (2.0%)	9100	202 (1.1%)	220 (1.2%)	18,425
Larva 51 hours	17 (0.19%)	121 (1.4%)	8828	17 (0.09%)	137 (0.72%)	19,029
Pupa 10 days	1392 (13%)	434 (4.2%)	10,404	2779 (12%)	581 (2.5%)	23,173
Adult 14 days	3194 (28%)	3093 (27%)	11,234	5167 (20%)	5953 (22.7%)	26,178

**Table 2:** Proportion of sex-biased genes and transcripts that show gonad-enrichment. Counts in the gonad columns show the number of sex-biased transcripts or genes that are differentially upregulated in testes (male-biased) or ovaries (female-biased). Counts in the Soma columns show the number of sex-biased transcripts or genes that are not differentially upregulated in testes or ovaries. Genes are counted as sex-biased if at least one of their transcription or splicing nodes is sex-biased.

Stage	Sex-biased Genes				Sex-biased Transcripts			
	Male		Female		Male		Female	
	Gonad	Soma	Gonad	Soma	Gonad	Soma	Gonad	Soma
Embryo 10 hours	4	22	6	139	4	25	6	168
Embryo 18 hours	6	182	1	184	6	196	1	219
Larva 51 hours	1	16	1	120	1	16	1	136
Pupa 10 days	260	1300	0	434	345	2434	0	581
Adult 14 days	411	2993	25	3032	609	4558	29	5924

Larvae show the least amount of sex-biased transcription (Table 1). Only one transcript (Nasvi2EG005321 or *Feminizer*) is sex-biased across all of development, followed by *Doublesex* (Nasvi2EG010980), which is female-biased in all stages from late embryo onward (>18 hours old). The low number of transcripts that are consistently sex differentially expressed across multiple stages is likely due to the low number of sex-biased transcripts in the pre-pupal stages. Transcripts that are differentially expressed in the embryonic stages are likely to be involved in sex-determination and will be of special interest for future studies.

Transcripts that shift between male to female-bias in different developmental stages are considerably less frequent than expected by chance (Fisher’s exact test, p-value ~0): only 508 transcripts, generated by 373 genes show this pattern (6% of all sex-biased genes in our final dataset). The majority (66%) of these transcripts display shifts from male-bias in pupae to female-bias in adults. Only 13 of these isoforms show gonad enrichment (1 of which is more expressed in ovaries and six of which are more expressed in testes). We find that 101 of the 609 transcripts enriched in pupal testes are female-biased at some stage in development (6%), and 16 of the 29 transcripts that are enriched in adult ovaries are male-biased at some stage in development (14%). Other patterns that include both male and female-bias across development consist of changes from female-bias in various pre-adult stages to male-bias in adults. Interestingly, transcripts with pre-pupal sex-bias are significantly more likely to show shifts between male and female sex-bias than transcripts with post-pupal bias only (Fisher’s exact test, p-value ~0). All transcripts described in this study and their annotations can be found in S1 File.

### Low relative prevalence of sex-biased isoform switching

**Fig 3:**
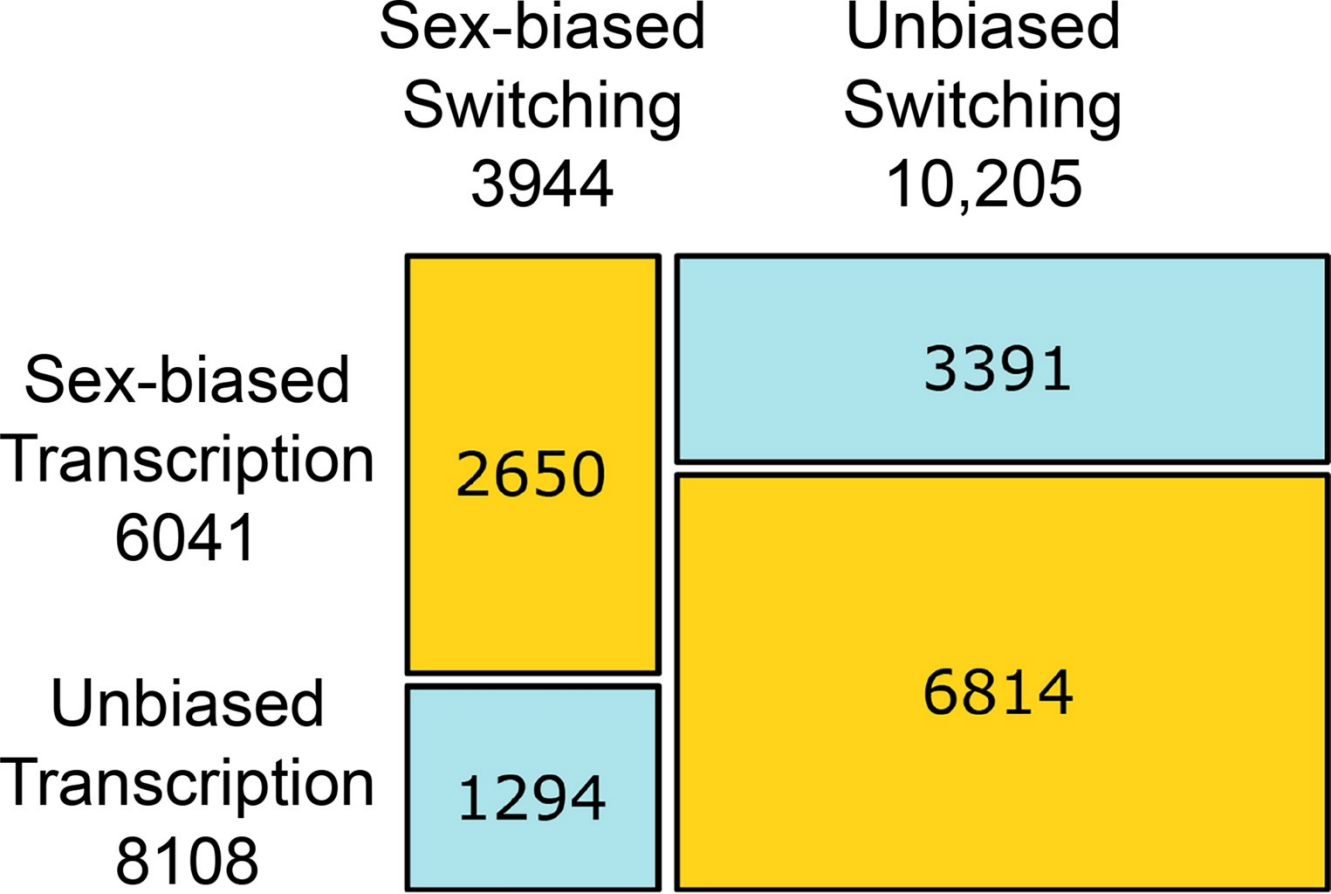
Proportion of genes with sex-biased isoform switching versus gene expression. The area of each rectangle is proportional to the total number of genes with the corresponding combination of sex-biased isoform switching (x axis) and gene expression (y axis). Numbers below each axis label report the total number of genes with sex-biased isoform switching or gene expression and numbers in each square report the absolute number of genes in each combination. Yellow cells indicate over-represented combinations, and blue cells under-represented combinations.

Genes with sex-biased gene expression are ~50% more frequent than genes with sex-biased isoform switching (6041 versus 3944). Over 67% of genes with sex-biased isoform switching also show sex-biased transcription, whereas less than 44% of genes with sex-biased transcription also show sex-biased isoforms (Fig 3). Only 1294 genes show sex-biased isoform switching alone, compared with 3391 genes with only sex-biased gene expression. Since only genes with detected isoforms can show sex-biased isoform switching, it is possible that the prevalence of sex-biased transcription might be caused by a large number of single-isoform genes. We thus repeated these analyses on the subset of genes that have at least one alternative isoform (8,194 genes), showing that the results remain unchanged (Fisher’s exact test, p-value ~0). Taken together, these observations indicate that bias in gene expression is the main determinant of transcriptome-wide differences between the sexes. Our estimates on the fraction of sex-biased adult *Nasonia* genes are consistent with those previously reported [21].

### Few genomic regions are enriched by sex-biased genes

We investigated whether regions of linked sex-biased loci are present in the *Nasonia* genome by searching chromosomal regions [50] for enrichment in male or female-biased genes. Current theories show that linkage could facilitate the evolution of adaptive sex-specific phenotypes by reducing the odds that alleles which work together are broken down by recombination [51].

Regions 1.065 and 5.072 show an enrichment for female-biased genes while region 4.1 is enriched in male-biased genes (Table 3). Female-biased group 1.065 contains *Nasonia*’s sex-lethal (Nasvi2EG000104) and histone deacetylase 3 (Nasvi2EG000106), a key component of histone-mediated gene regulation.

**Table 3:** Linkage groups enriched by sex-biased genes. Numbers indicate gene counts with their percentages compared to all genes in the linkage group. Recombination rates are expressed as centiMorgan per Mb. The last row reports median proportions and recombination rate across all linkage groups.

Linkage Group	Enrichment	Male-biased Genes		Female-biased Genes		Total Genes	Recombination Rate
4.1	Male	49	45%	12	11%	109	0.093
1.065	Female	3	8.3%	18	50%	36	0.38
5.072	Female	12	8%	59	40%	147	0.086
Median Values	None		17%		17%		0.25

Female biased linkage group 5.072 is strongly enriched by the controlled Gene Ontology (GO) annotation terms “apoptosis of nurse cells” (GO:0045476) and several other developmental terms related to photoreceptor and neuronal development (e.g. brain morphogenesis). Most genes within the male-biased linkage group 4.1 belong to cysteine-rich secretory proteins (PF00188.21). While these proteins are currently annotated as venom allergens, we hypothesize that the same secretory domains may, in this case, be involved in sperm production, as is suggested by expression patterns of their homologs in *Drosophila* [52].

Overall, the male enriched linkage group accounts for 1.2% of male-biased genes. The female-enriched linkage groups account for 2.0% of female-biased genes. While theory predicts selection for lower recombination rates in sex-biased genomic regions [53,54], recombination rates in all three linkage groups fall within the interquartile range of recombination rates of all linkage groups.

### Switches in sex-bias identify meiosis genes

To provide a better overview of gene expression patterns, we grouped transcripts in co-expressed transcriptional clusters (henceforth **clusters**) and tested their average expression for significant sex-biased differential expression (**DE**). The results of these analyses at the cluster level are quantitatively similar to the results obtained by examining single transcripts independently. Out of the 172 clusters of the whole transcriptome (Fig 2B), 36 are male-biased clusters (6489 genes), seven clusters are enriched by testis-biased genes (2552 genes, 40% of all male-biased genes) and 41 clusters are female-biased clusters (7444 genes), two of which are enriched for ovary-biased genes (698 genes, 9% of all female-biased genes). The full assignments of transcripts to clusters are reported in S1 File, and the results of cluster-level analyses are reported in S2 File. Clusters are labeled as colour names, by convention [36].

We find four clusters that alternate between male and female-biased expression in different developmental stages (**Fig 2B**). Cluster ‘green3’ shifts from male-bias in late embryos to female-bias in larvae. Its members consist of retro-transcriptases and unannotated multi-copy genes. The observed sex-bias in this cluster is thus most likely related to the activity of transposons and unlikely to serve a functional role in the development of sexual characteristics of individuals.

The remaining three clusters (‘antiquewhite4’, ‘lightpink2’ and ‘yellow4’) shift from being male-biased in pupae to female-biased in adults. Clusters ‘antiquewhite4’ and ‘yellow4’ are enriched by meiosis and gametogenesis related gene ontologies. Cluster ‘lightpink2’ contains several genes coding for amino acid binding proteins, including condensin (Nasvi2EG004100), which is involved in chromosome assembly and segregation [55].

Since *Nasonia* male gametogenesis occurs during the pupal stage, while female gametogenesis occurs primarily during adulthood [49], the shift in sex-bias observed in these clusters is likely caused by differences in the timing (heterochrony) of activation for gametogenesis-related genes. When we compared the top-ranking hubs in each of those clusters with their Wasp Atlas entries [56], we discovered that other studies reported these to be moderately to extremely testis-biased in *Nasonia* [57] thereby supporting the hypothesis that these clusters are primarily involved in male gametogenesis.

### Differential correlation reveals early sex-biased transcription

**Fig 4:**
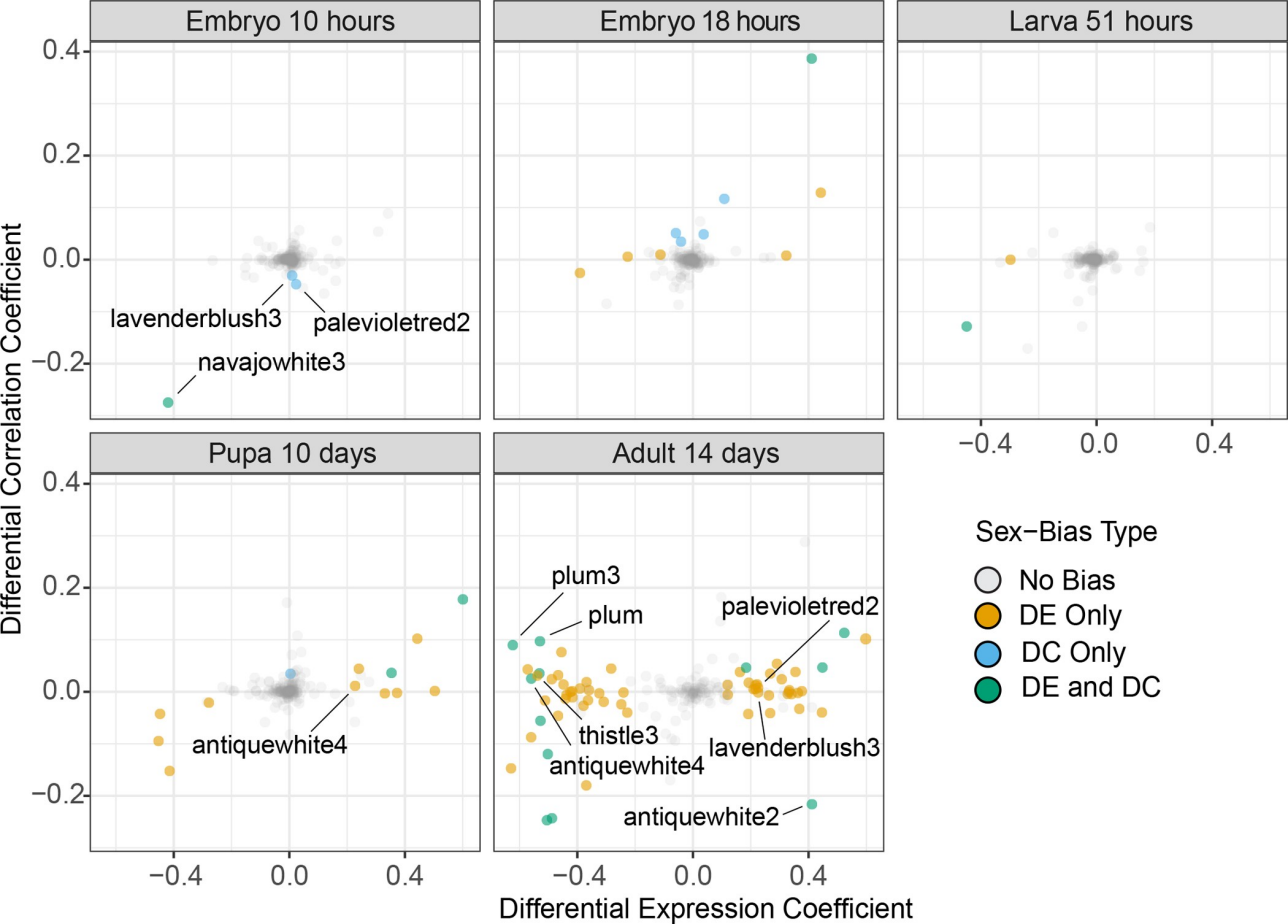
Cluster-level sex-biased expression and correlation across development. Each dot represents a single transcriptional cluster in a single developmental stage. Position indicates the degree of sex-biased expression (x axis) and correlation (y axis). Positive values indicate male-bias, negative values indicate female-bias. Clusters are coloured in accordance with significant differential expression or differential correlation (see Methods).

Our differential correlation (**DC**) analyses detected several clusters that show significant sex-biased differences in transcript-transcript correlation (co-expression, or gene-gene interactions) within any of our five developmental stages, regardless of whether the cluster is also differentially expressed (**DE**) between the sexes (see Materials and Methods for details). This analysis revealed 65 clusters of differentially correlated genes (Fig 4). Most DC clusters confirm the DE findings, with the notable exception of embryonic stages: six of eight clusters with DC in embryos show no significant DE. In other words, a majority of early transcriptomic differences between the sexes are revealed to be relevant in the development of females *versus* males only when considering gene-gene interactions, rather than individual gene expression levels.

Only one of the DC clusters in early embryos is also DE (cluster ‘navajowhite3’) showing increased expression in female early embryos. This cluster is enriched by several gene ontologies related to nucleosome assembly, which include both the histone acetyltransferase complex H4/H2A HAT and several histones that and are likely to have undergone a lineage-specific gene family expansion [58]. We therefore speculate that this cluster could be involved in sex differences mediated through histone modifications or chromatin condensation, and that genes in the cluster could be of special interest to future studies on the regulatory mechanisms involved in sex-determination.

Two additional clusters (‘lavenderblush3’ and ‘palevioletred2’) show no DE in early embryogenesis, but do show female-biased DC at that stage. Both are also differentially under-expressed in adult females relative to males. Why this pattern occurs is unclear, but could be indicative of early sex determination processes. Neither cluster shows enrichment in informative gene ontologies. The most connected genes in these clusters show strong testis enrichment (i.e. Nasvi2EG018256 and Nasvi2EG007678, [57]) suggesting that that these clusters may be involved in spermatogenesis, although this does not explain why they also show DC in early female embryos.

Only one cluster (darkseagreen2) shows significant male-biased DC in early embryos. This cluster contains 75 genes and is strongly enriched by gene ontologies related to stem-cell fate determination, neurogenesis and down-regulation of RNAs. We are uncertain about the potential role of these genes in very early sex differentiation, which may reflect timing differences in male and female early development that are detected as male-biased DC in early embryos.

While the direction of sex-bias is generally consistent between differential correlation and differential expression, we find that five of the 20 clusters with simultaneous DE and DC show bias in opposite directions. All of those exceptions are observed in adults. Four of these clusters (‘antiquewhite4’, ‘plum’, ‘plum3’ and ‘thistle3’) are more highly expressed in adult females, yet more strongly correlated in adult males. The fifth (‘antiquewhite2’) is more highly expressed in males yet more strongly correlated in females.

An increase in mean expression could affect the analysis of differential expression but not differential correlation. This may specifically apply to cluster ‘antiquewhite4’, which (as mentioned earlier) is likely to be involved in gametogenesis. We observe enrichment for gametogenesis associated annotations in the cluster ‘plum3’, and germ-cell development for cluster ‘thistle3’. All hub genes of these clusters (and of cluster ‘plum’) show moderate testis-bias in adults of *Nasonia* [57]. Gene ontologies of cluster ‘antiquewhite2’ include signal transduction, and its hub contains several isoforms of Nasvi2EG010141, a calcitonin receptor enriched in female heads [59]. However, we find that this cluster is enriched in testis-biased genes in our experiment, suggesting that it might play different roles in different sexes and tissues.

### Sex-biased clusters have different regulatory structure

**Fig 5:**
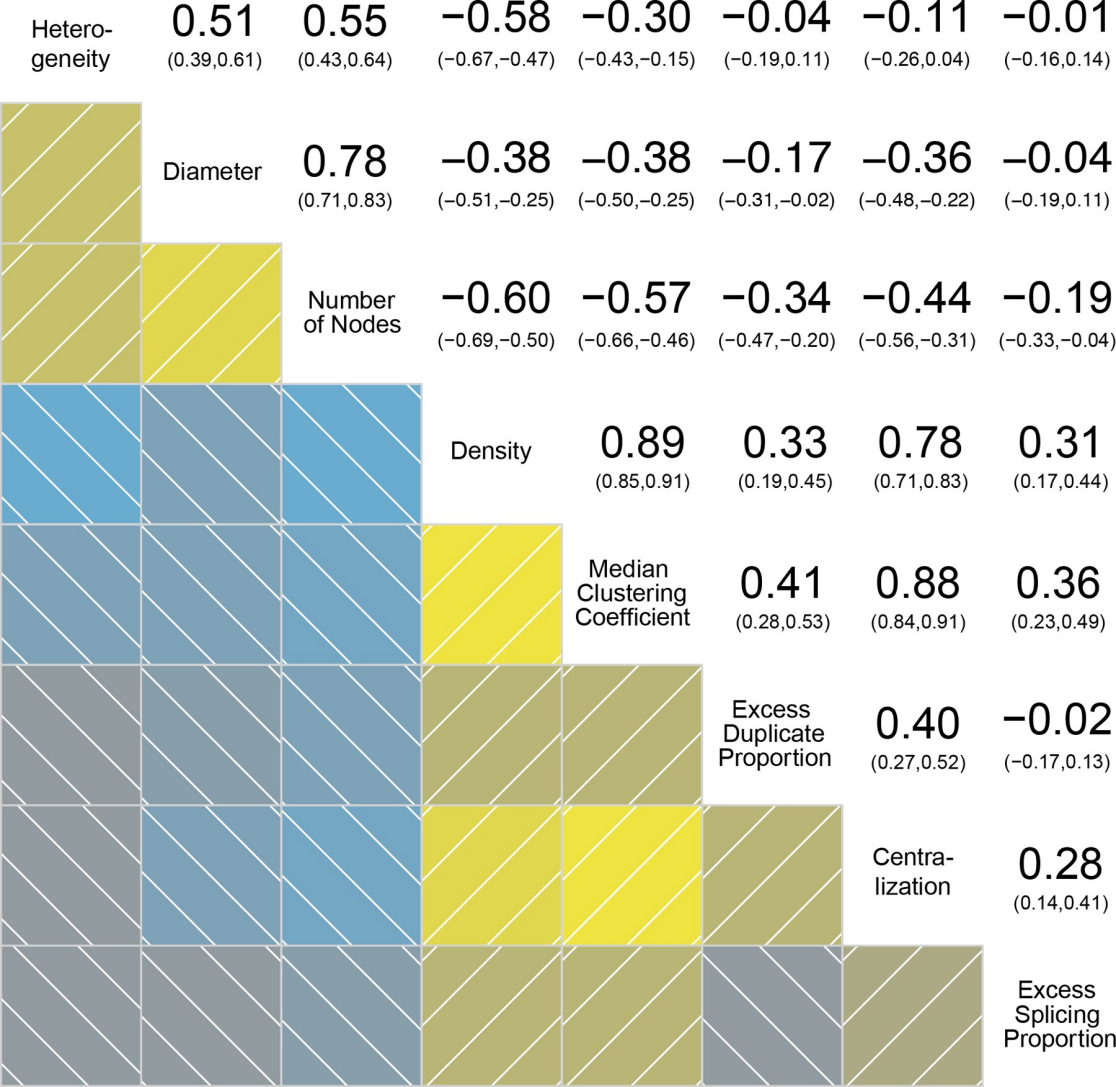
Correlations between pairs of topological network parameters. Network parameters are annotated on the diagonal. Yellow squares in the bottom left corner indicate positive correlations, blue squares negative correlations. Lighter shades are more significant than darker ones. Numbers at the top right corner indicate the Pearson correlation score with confidence intervals in parentheses.

Because each cluster is a network of co-expressed transcripts, we investigated whether the clusters that are sex-biased have different regulatory structures compared to clusters that are unbiased–by characterizing the network architecture of each cluster (see network structure variables in the Materials and Methods). Several topological network parameters suggest that these sex-biased clusters are composed of non-trivial associations among nodes (see Fig 5). We therefore carried out a principal component analysis (PCA) to delineate the features (Principal Components, or PCs) that best separate networks. We then tested whether sex-biased clusters show significant differences across these PCs.

**Fig 6:**
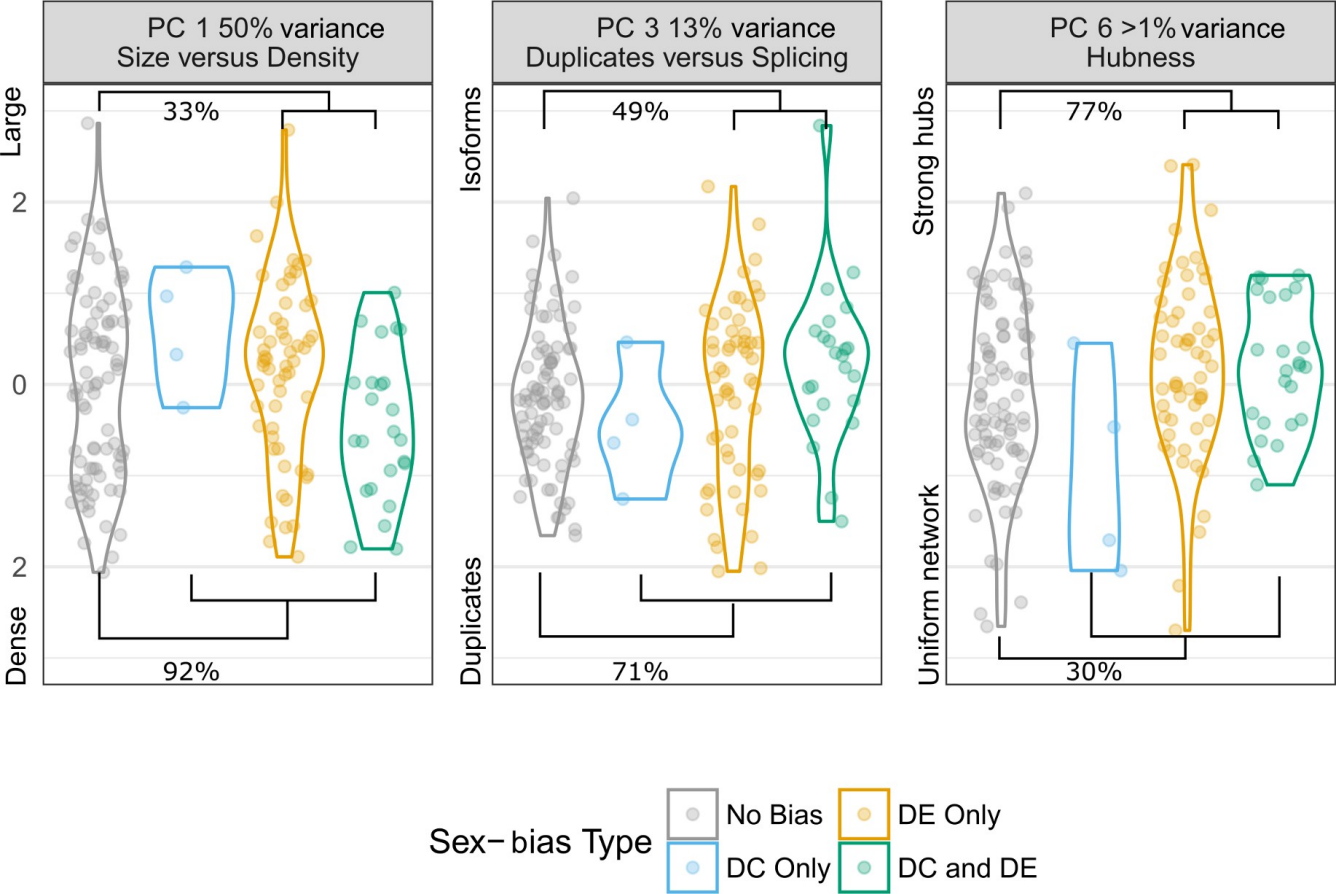
Topological differences between sex-biased and non-sex-biased clusters. Percentages in each panel show the probability (RI) that a parameter differs between clusters with no sex-bias and clusters with differential expression (top of the figure) or differential correlation (bottom of the figure, see Methods for details) between sexes. Each of the three panels show one of the PCs that describes each cluster, with the percent of variance explained. The left panel (PC 1) discriminates between small clusters with many connections (negative values) and large clusters with few connections (positive values). The middle panel (PC 3) discriminates between clusters with many duplicated genes, but few genes with isoform switching (positive values) and clusters with few duplicated genes but many genes with isoform switching (negative values). The right panel (PC 6) discriminates between clusters with a uniform distribution of connection (negative values) and clusters with few genes with many connections and many genes with few connections (positive values).

To characterize DC clusters, we contrast clusters with no form of sex-bias with clusters that are differentially correlated at any one stage of development, regardless of whether they also show DE. Likewise, DE clusters are defined here as clusters that show DE at any one developmental stage, regardless of the presence of DC. Three PCs of cluster architecture (PC 1, 3 and 6) are significantly different (Relative Importance or RI >70%) between sex-biased and non-sex-biased clusters (Fig 6).

The strongest association (RI 92%) we observed is between differentially correlated clusters and PC 1. PC 1 is also the only factor that significantly discriminates between clusters with sex-biased expression and clusters with sex-biased correlation (RI 93%). The lower scores of differentially correlated clusters on PC 1 indicate that they tend to have fewer genes with strong connections between them. Differentially correlated clusters are also moderately associated (RI 71%) with PC 3, which indicates that they have more duplicates and less isoforms than unbiased networks. This finding is in accordance with theories and empirical observations on how gene duplication can solve sexual conflict at the gene level [16,60–63].

While PC 6 explains less than 1% of between-cluster variance, it is the only PC that is significantly associated with differentially expressed clusters (RI 77%). The high PC 6 scores of differentially expressed clusters indicate a more hierarchical structure, with a stronger divide between hyper-connected regulatory hubs and peripheral worker nodes (see Methods for full description). This result suggests that differentially expressed clusters have a more ‘star’ shaped topology, in which most nodes are connected to hubs but not among each other.

### Sex-biased clusters integrate new genes in regulatory positions

**Fig 7:**
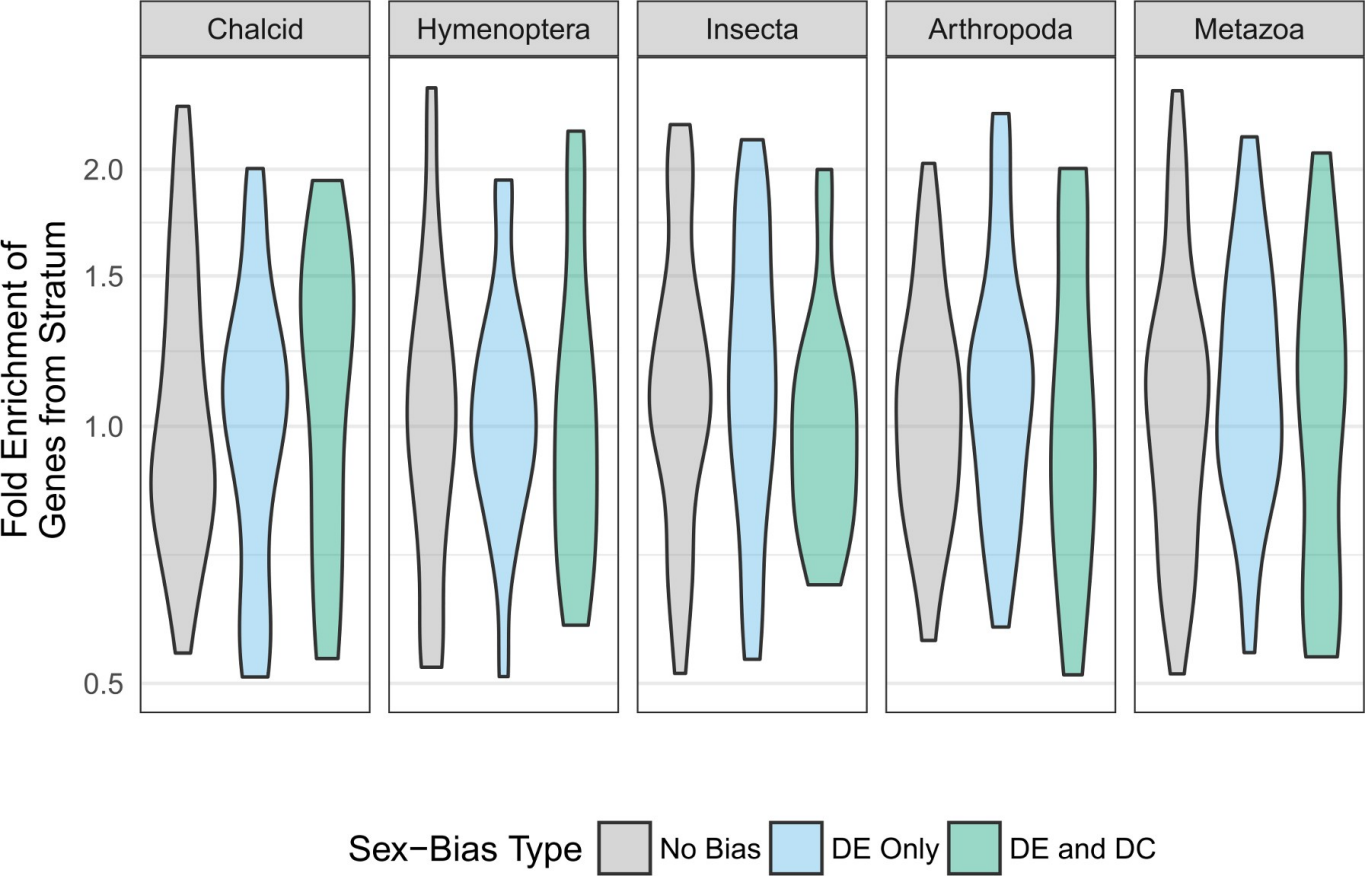
Fold enrichment of genes form different ages for clusters with and without sex-bias. Each panel shows the proportion of genes of a specific age in clusters with different types of sex-bias, compared to genome-wide averages (fold enrichment). Gene age is measured as the highest taxonomic level required to describe all organisms that have homologs for the gene (oldest common ancestor, see Methods). Clusters are divided between those with no evidence for sex-bias (grey), those with evidence only for differential expression (blue) and those with evidence for both differential expression and differential correlation (green). Positive y values indicate that the clusters contain more genes of a specific age compared to genome-wide average (enrichment), measured in relative fold-change. For instance, a value of 1.5 indicates that the cluster contains 50% more genes of that age than expected from genome-wide average.

To validate whether sex-biased clusters show more rapid evolution compared to non-sex-biased ones, we initially compared the times of phylogenetic origin (stratum) of genes in both categories using data from [64]. Reference species include non-chalcid hymenopterans (seven ants and five bees), insects, arthropods, and other metazoans (Fig 7, and see materials and methods for full species list). Compared with non-sex-biased clusters, sex-biased clusters show a greater proportion of genes that originate at the youngest taxonomic level (here chalcid). Among sex-biased clusters, those with both sex-biased differential expression and differential correlation (DE+DC) show a slightly higher proportion of chalcid-stratum genes compared with clusters with only sex-biased differential expression (DE).

We note that genes apparently absent in older phylogenetic strata are either relatively recently evolved genes or genes that evolve sufficiently rapidly that their homology to other taxa have been obscured. For convenience, we refer to these here as “new” genes. Compared to clusters that show only DE, DE+DC clusters appear depleted of genes from more ancient phylogenetic origins (or strata), such as Hymenoptera, Insecta and Metazoa.

**Fig 8:**
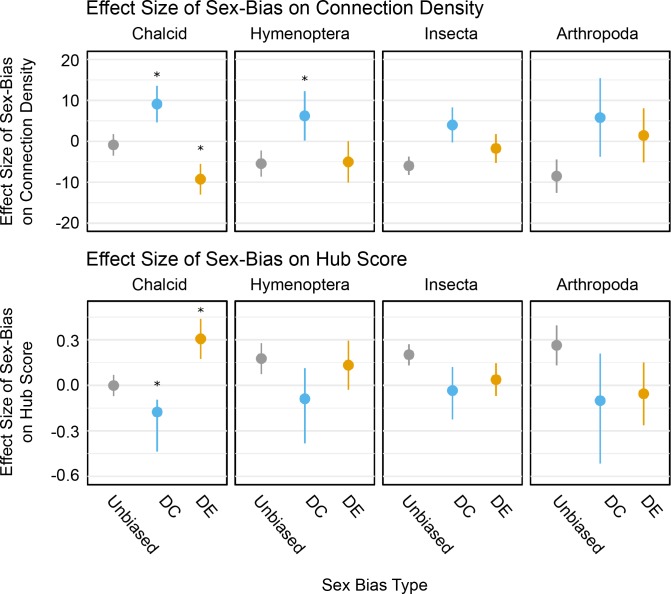
Topological properties of regulator, interactor and marginal nodes. Nodes represent individual transcripts; connections represent correlations in expression values. Transcript A (regulator) connects to several other transcripts (high connection density) which are not connected between each other (high hub score). Transcript B (interactor) connects to several transcripts (high connection density) which are connected between each other (low hub score). Transcript C (marginal) connects to few nodes (low connection density) which are already connected between each other (low hub score).

We next addressed whether new genes in sex-biased clusters are more likely to be in regulatory positions than new genes in non-sex-biased clusters. We compared the impact of gene age (phylogenetic stratum) with two main network properties: within-cluster connection density and hub scores. Connection density measures the number of interactions between the gene of interest and other members of its cluster. Hub scores estimate the regulatory potential of the gene of interest within its cluster (see Methods section and **Fig 8** for details).

**Fig 9:**
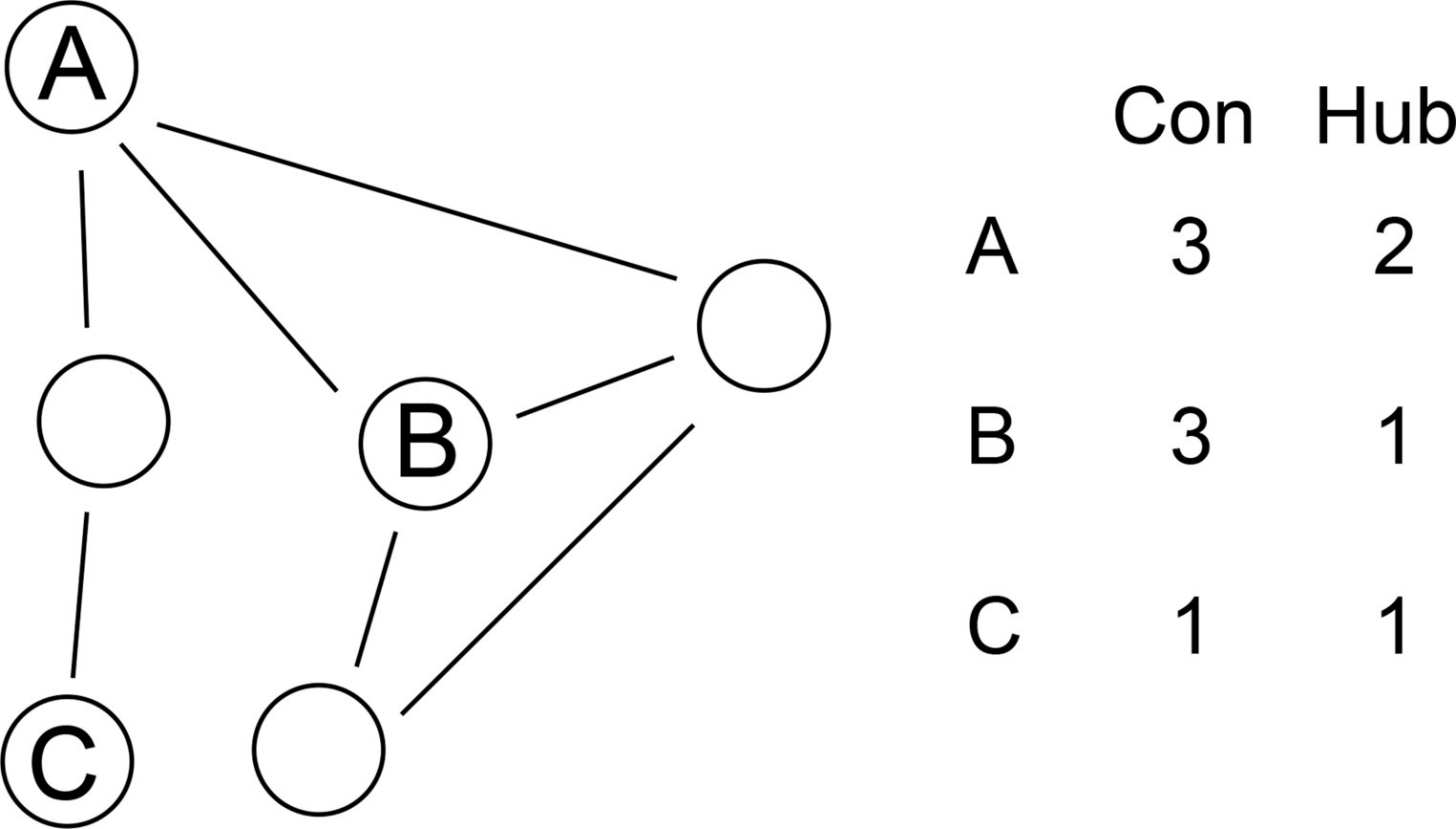
Topological properties of sex-biased transcripts of different ages. Each panel shows the average difference in connection density (top row) and hub score (bottom row) of transcripts from a specific age class compared to transcriptome-wide averages, with 95% confidence intervals. Positive scores mean that the clusters have higher connection density or hub scores compared to metazoan-level genes of the same sex-bias type (see Methods for details). Colours show which transcripts belong to clusters with sex-biased expression, sex-biased correlation or no sex-bias. We annotate clusters as DC regardless of whether they have DE, and vice versa. Contrasts marked with an asterisk indicate that transcripts in clusters with sex-biased expression or correlation have different connection density (top row) or hub score (bottom row) than transcripts in unbiased clusters for that age class. See S4 File for full model results.

We tested whether either of these two parameters are associated with the gene’s age (stratum), and whether the gene is part of a sex-biased cluster, as well as the interaction between gene age and sex-bias (Fig 9 and S4 File). Since DE and DC clusters show different topologies (see previous section), we analyzed them as separate classes of sex-bias. In this analysis, we annotate as DE all clusters that show sex-biased differential expression, regardless of their DC status and vice versa. We chose to also include cluster size as a predictor, since it strongly correlates with several features of overall cluster topology (see previous section and Fig 5), thereby controlling for potential interference from cluster-wide effects.

We find that cluster size has significant effects on both density and hub scores (RI 100%), but in both cases, effect sizes are an order of magnitude smaller than those of gene age and sex-bias. Gene age is strongly associated with both connection density and hub scores, as indicated by its high relative importance in both models (RI 100%). Overall, genes from intermediate strata (Arthropoda, Insecta, and Hymenoptera) show lower connection densities and higher hub scores than younger genes, suggesting that genes from intermediate strata are most frequently located in positions with high regulatory potential within their clusters. This pattern is reversed for genes assigned to the oldest stratum (Metazoan), which show high connection densities paired with low hub scores. This latter configuration is characteristic of interactor nodes (see Methods), which function by closely interacting with each other, rather than by directly regulating downstream elements. Accordingly, Metazoan stratum nodes are enriched by protein complexes (GO:0043234, q-value ~3.1 *e*^*−*96^) such as flagellar proteins in clusters ‘tomato’ and ‘skyblue3’, and spindle formation in cluster ‘thistle’ (S1 File).

Genes in DC and DE clusters show a different relationship between age and topological properties than genes in non-sex-biased clusters. Genes in DC clusters show a decrease of connection densities with increasing gene age compared with non-sex-biased clusters (RI 100%), but maintain the overall pattern of increased hub scores with gene age (RI 23%). The position of young genes in DC clusters is thus consistent with that of interactor nodes (see Methods), with low regulatory potential. By contrast, the relationship between gene age and topological properties is reversed for genes in DE clusters; younger genes show both decreased connection densities (RI 100%) and increased hub scores (RI 95%). This distribution indicates that younger genes in DE clusters conform to the expectation of hubs (see Methods), which bridge connections between otherwise independent group of genes and enable coordinated regulation.

To more precisely characterize the age of the young regulators in sex-biased clusters, we integrated phylostratigraphic assignments from additional parasitoid wasp species [65] in the superfamily Chalcidoidea. In addition to *Nasonia* (family Pteromalidae), the chalcids used here are those with published genome assemblies, and belong to different chalcid families, *Ceratosolen solmsi (Agaonidae fig wasps)*, *Copidosoma floridanum* (Encyrtidae), and *Trichogramma pretiosum*, in the more basal family Trichogrammatidae. The most recent common ancestor of these parasitoids is estimated to be approximately 99 MYA [66]. These data further separate genes from the hymenopteran stratum into genes that are shared among the chalcids (but not the other hymenopterans–Chalcid stratum) and those that are unique to *Nasonia* but not found in other chalcids (*Nasonia* stratum). The split between the *Nasonia* clade and its closest chalcid among our set (*Ceratosolen solmsi*) is approximately 71 MYA, followed by *Copidosoma floridanum* at ~81 MYA [66]. Even after using this finer scale, only *Nasonia*-stratum genes show increased connectivity and hub scores in DE clusters relative to hymenoptera-stratum genes (S5 File), indicating that these features have evolved in the ~70–80 million year span that separates *Nasonia* (family Pteromalidae) and the most closely related Chalcid wasps included in our analyses [66,67]. While we did not detect the same pattern for DC clusters at this level of analysis, this is most likely due to the low number of genes with DC patterns that are assigned to the Chalcid stratum.

## Discussion

Our assessment of sex-biased gene regulation across the development of *N*. *vitripennis* leads to several discoveries. We find relatively few differentially expressed (DE) genes early in development (early embryo, later embryo and larva). In contrast, four clusters show early sex-biased differential correlation (DC). These DC clusters contain another category of genes that can be important in early stage sex determination and differentiation but would not otherwise be detected by differential expression techniques.

We see a dramatic increase of DE genes in the pupal stage, when considerable sex differential development is occurring, which continues into adulthood. At the gene level, we observe a prevalence of sex-biased gene expression relative to sex-biased isoform switching. Furthermore, genes are generally consistent in their sex bias during development. We find only a small number of genes that shift between male and female-bias across developmental stages. Most of the genes that switch between male and female bias may be attributed to differences in the timing rather than function between sexes (e.g. gonad maturation occurs earlier in males than females). We also identify several genomic regions enriched in male and female-biased genes, although their biological significance remains unclear.

At the cluster level, we report two distinct types of sex-bias with specific temporal expression patterns and topological properties. We detect differential correlation early in development, in clusters that are small and with many interactions. Differential expression instead becomes prevalent only during pupation, and occurs in networks which have young or fast-evolving genes in potentially regulatory positions. Both DC and DE cluster genes provide reasonable candidates for future studies on sex-differentiation within the hymenopteran lineages.

### Prevalence of whole-gene transcriptional bias

Our analyses of differential expression suggest that regulation of whole-gene transcriptional level may be the most frequent molecular process inducing transcriptome-wide differentiation between the sexes. We find far more loci with evidence of sex-biased gene expression than sex-biased isoform switching. More importantly, the majority of genes with sex-biased transcription do not show sex-biased isoform switching, whereas most genes with sex-biased isoform switching also show sex-biased transcription, suggesting that isoform switching might be mostly redundant. This finding is consistent with studies in *Drosophila* development [27], which show that the majority of isoform variation is observed either between tissues or between stages, and that the few consistently sex-specific isoforms in adults can be attributed to sex-specific tissues.

Nonetheless, we describe 1,294 (~10% of 14,149) genes that show sex-biased isoform switches and lack sex-biased gene expression. This finding doubles the reported frequency in adult *Drosophila* [27], yet is consistent with earlier *Drosophila* estimates from studies aiming at the specific detection of sex-biased alternative splicing [25,26]. It is noteworthy that [27] measured transcript expression via RNA-seq technologies whereas both earlier *Drosophila* studies and our study relied on microarrays. As such, more molecular data are required to validate our findings on the scope of sex-biased isoform switching.

Despite the abundance of sex-biased transcripts, only one gene (feminizer = *transformer*) shows consistent sex-bias across all developmental stages, while the majority of sex-bias is observed in either the pupal or adult stages. A study comparing pre-gonad imaginal discs in larvae and pupae to adult gonads in *Drosophila* found that 50–60% of sex-biased genes retain their expression-bias across larval, pre-pupal and adult stages [22]. However, that study is not comparable to ours, since we compare whole body expression from early embryo to adult stages. Our estimates are closer to those observed in a study of chicken [23], which includes embryonic stages but again is focused on gene expression in gonads as opposed to the whole body study conducted here. The broad pattern is also potentially in accord with observations for caste-specific genes in ants [68].

We find three main genomic regions enriched by sex-biased genes. But there is no evidence of reduced recombination, counter to the expectation of co-evolution driven linkage [51,69]. Co-expression of closely related genes may arise as a side-effect of tandem duplications, which can generate a large number of neighboring genes that share expression pattern because of identity by descent. This seems to be the case for the male-biased linkage group 4.1, in which a series of tandem duplications for male-biased “venom allergen” proteins (orthologous group EOG8W9MM2) is present. Since male *Nasonia* do not produce venom, we find it more likely that these proteins are a component of male seminal fluids, a function that has indeed been reported for their *Drosophila* homologs [52] and would be consistent with the presence of secretory domains.

### Heterochrony in gametogenesis drives developmental sex-bias shifts

Only four clusters show shifts between male and female-bias at different developmental stages, suggesting that developmental sexual conflict significantly constrains sex-biased gene expression. After excluding one cluster composed mostly by transposons, the remaining three clusters include 324 genes (1.1% of the whole network). All three clusters shift from male-bias in pupae to female-bias in adults. These directional shifts in sex-bias may be explained by the timing of *Nasonia*’s gametogenesis. *Nasonia* spermatogenesis peaks during pupation, while its oogenesis occurs primarily during the adult stage [40]. As such, we expect that genes required for gametogenesis will be expressed during pupation in males and during adulthood in females. The change in sex bias from male to female after pupation could thus be caused by gametogenesis genes that are not yet expressed in females during pupation, or have already been “switched off” in adult males. Genes in these male-to-female clusters are thus most likely performing similar functions at different times (heterochrony) rather than different functions in each sex.

While we identify several genes with female to male shifts in sex-bias (see S1 File for details), this expression pattern is not represented in any cluster, suggesting that these genes may be outliers rather than members of functional sub-networks. These patterns contrast with those found for some other insects. By comparison, *Drosophila* shows sex-bias shifts in development in 4.9% of autosomal genes and 2.9% of X-linked genes [22], while a study on *Bombyx mori* reports them for 54% of its transcriptome [24]. In both cases, the observed shifts are primarily (*Drosophila*) or exclusively (*Bombyx*) from female-bias in early stages to male-bias in the latter ones. However, these studies concerned changes in expression in pre-gonadal versus gonadal tissue (*Drosophila*) or the larval-pupal transition (*Bombyx*).

We cannot rule out that the increase of female expression in adults may be due to the greater proportional mass of gonads present in adult females compared to males. Characterization of clusters that show female-biased DE and male-biased DC reveals enrichment in testis-related processes, suggesting that (at least in these cases) tissue-bias in adults is strong enough to reverse measured gene expression-bias. Likewise, the low number of ovary-enriched genes detected in the females could also be caused by the high proportion of ovarian tissue present in the females. Since we contrast whole-organisms with gonads, a high representation of ovarian tissues in adults will result in lower mean differences in gonad to adult contrasts, decreasing our power to detect genes with low but significant differences in expression. To the best of our knowledge, only one study to date separated gonads from the carcasses in males and female *Nasonia* before RNA sequencing [70]. While their findings are similar to those obtained by whole organism sequencing in [21], the study focused on genes with at least 100-fold expression differences–effectively pre-selecting only for sex-specific genes rather than sex-biased ones.

### Sex-bias in early development

We identified several early regulatory events using complementary analyses based on both differential expression (DE) and correlation (DC). Embryonic stages in particular show little differentiation between sexes when relying exclusively on differential expression. Yet several co-regulatory events are revealed by the differential correlation analysis. For instance, only one cluster containing 79 genes is differentially expressed in early female embryos, compared to three differentially correlated clusters containing a total of 373 genes. Differential cluster expression identifies no male-biased genes in early embryos, whereas differential cluster integration reveals 75 genes in male-biased clusters.

Among the clusters with female-biased correlation in the early stages, two are also differentially under-expressed in adult females relative to males. This pattern is potentially indicative of paternal imprinting mechanisms that influence early development. However, sperm is already mature in adult males, and therefore the opportunity for genomic imprinting is passed. It is possible that effects are mediated through seminal fluids. An alternative is that the clusters reflect induction of developmental pathways in early embryos derived from fertilized eggs, which are destined to become females. In *Nasonia*, male appears to be the default sex, with feedback between maternal derived and zygotic *doublesex* being required for induction of female development [28,71]. Under-expression of these genes in the adult female (e.g. ovaries) could reflect a male default pattern set in the eggs, with induction of the female developmental cluster through *doublesex*. Therefore, the genes in these DC clusters may be important for this early sex differentiation.

Histone and histone-modification enzymes are enriched and occupy hub positions in the only early embryonic cluster that also shows both DE and DC sex-bias. Overexpression (DE) of histones in diploid females may be expected due to the greater amount of DNA in their cells but DC is caused by female-specific increase in expression correlation, which is independent from absolute expression. The presence of DC suggests that histones and their modification enzymes may be involved in sex-specific interactions in early embryogenesis. This result is especially interesting in light of the ongoing debate on *Nasonia*’s sex-determination mechanism. While there is now a consensus on the need for a silencing mechanism of maternal Feminizer expression [13,28], the specific mechanism has so far been elusive. Several papers aimed at investigating the role of DNA methylation have shown that genes subject to DNA methylation show less variation across evolutionary and developmental space [72,73] and there is very limited evidence for sex-biased differential methylation in adults [21]. Our study reinforces a lack of support for DNA methylation as a mechanism for sex-biased genome imprinting, suggesting instead the modification of specific histones as a possible alternative. Since the genome copy carried by sperm is bound by sperm-specific protamines [70] rather than histones, such a mechanism would provide a robust means of erasing only paternal imprinting without the need for divergent histone markings in adults. Histone-mediated *Nasonia*-specific control of sex-determination would also be consistent with our earlier finding that histone gene families are specifically expanded in *Nasonia* relative to ants and bees [58], and that some of those members are indeed part of the clusters with early sex-bias.

### Network structure of sex-biased clusters

Sex-biased clusters show high proportions of *Chalcid*-stratum genes (Fig 7), which occupy different positions within their networks (Fig 9). In differentially correlated clusters, *Chalcid*-stratum genes are highly connected but have low hub scores, suggesting that younger (or more rapidly evolving) genes have low regulatory potential. In differentially expressed clusters, the younger (or more rapidly evolving) *Chalcid*-stratum genes are instead sparsely connected and show the highest hub-scores, consistently with the expected topological properties of regulators. The enrichment of *Chalcid*-stratum nodes in hub-like positions in DE clusters is higher than expected when compared to both DC and non-sex-biased clusters. While the imputation of causal molecular roles from gene correlation data remains subject to debate [74], this result suggests rapid evolution of sex-bias networks in DE clusters relative to more stable non-sex-biased networks. However, studies that include stronger prior knowledge of the *Nasonia* gene regulatory network [75] and validation via gene-editing are required to properly assess the topology of sex-biased networks.

The method of phylostratigraphic dating can also generate biases [76], particularly when attempting to detect deep matches for short or rapidly evolving genes whose sequence similarity rapidly degenerates below homology criteria. Considering that sex-biased genes have indeed often been observed to have faster evolutionary rates [21], it is likely that a portion of *Chalcid*-stratum genes will consist of rapidly diverging genes from older strata. Depending on the extent of phylostratigraphic-bias, we can interpret these findings in two ways. Either new genes are indeed more readily integrated in key regulatory positions within differentially expressed networks (low phylostratigraphic-bias scenario) or genes in key positions of sex-biased networks in the *Nasonia* clade have rapidly mutated beyond homology criteria (high phylostratigraphic-bias scenario). Both scenarios imply that genes in DE clusters show significant evolutionary differences compared to non-biased ones, and that those differences are correlated with the genes’ positions within the regulatory network. Rapid integration of novel genes into regulatory positions of sex-specific networks has already been documented multiple times in *Drosophila* for mechanisms as diverse as male fertility [77,78] and courtship specificity [78], whereas over 75% of the caste-biased genes in the social wasp *Polistes canadensis* lack homology outside of the species [79].

### Conclusion

Here we present an investigation of sex biased gene expression and co-expression from early development (embryo) to adulthood in an insect. *Nasonia*’s sexual development reveals numerous interesting properties about the evolution of sexual dimorphism and identifies sets of candidate genes for early and stage-specific sex differentiation that provide further understanding on the evolution of sex-determination in Hymenoptera.

We also make a first detailed comparison of the interplay between gene expression and isoform switching over *Nasonia’s* sexual development, and note that sex-biased isoform switching is in most cases present in loci that already show sex-biased gene expression. While the overall results point to a prevalence of gene regulation over isoform switching as a means of inducing sex-biased differences, we also annotate isoform switch events that occur in loci without sex-biased gene regulation and are thus theoretically capable of inducing differences between sexes.

Our analyses of early developmental expression reveal that differentially correlated sets of transcripts could play a role in the onset of insect sexual differentiation and possibly sex-determination itself. Despite the lack of genetic sex-determination, we find at least three genomic regions enriched in sex-biased clusters. Several scenarios can explain their presence, spanning from selective advantage of their co-inheritance to non-adaptive linkage hitchhiking and tandem duplication. Discriminating between these options will require modelling that integrates knowledge about *Nasonia*’s genome with its ecology and taxonomy. *Nasonia*’s sex-bias is strongly developmentally restricted, with few transcripts showing sex-bias in multiple stages. While several genes show male to female sex-bias changes between stages, they belong mostly to genes with different timing between sexes or contrasts between pre- and post-pupation stages.

Finally, our network analyses provide an initial overview of the role of genes of unknown function within sex-biased clusters and points to potential candidates for future studies on how new genes can contribute to sex-specific differences. More generally, because gene ontologies for most insects are derived from homology to genes of known function in *Drosophila* and other model organisms [80], many genes in other insects can be of uncertain ontology. Differential Correlation clustering can provide predictions about function of such genes due to their associations with those with gene ontology annotation, thus providing a tool for investigating function of novel or rapidly evolving genes with obscure gene ontology under current methods.

## Materials and methods

### Biological materials and data collection

The data for this study consisted of a developmental time series of transcriptomes occurring in whole animals, both for male and female jewel wasps (*Nasonia vitripennis*). Our experiment was conducted using a highly-inbred strain called AsymCX [46]. Inbreeding occurs routinely in *Nasonia* without the deleterious effects often found in outbreeding diploid species, primarily due to purging of mutational load in the haploid sex [81] and provides a reduced level variation due to genetic differences between individuals.

We sampled animals at five distinct developmental stages: early embryo (0–10 hours old), late embryo (18–30 hours old), 1^st^ instar larvae (~51 hours old), yellow pupa stage (~14 days old) and sexually mature virgin adults. Specifically, samples taken during the first embryonic stage were of single zygotes up to late blastoderms, prior to gastrulation. The samples taken during the late embryonic stage were of embryos that exited gastrulation and include segmentation, organogenesis and the remaining pre-hatching development (for reference timings, see [82]).

Each of these developmental stages was sampled in triplicate for each sex, for a total of 30 samples to profile. Because of the different number of cells at different stages, different numbers of sampled individuals were pooled for each biological replicate as follows: 300–900 individuals for early embryos, 140–500 for late embryos, 245–520 for 1^st^ instar larvae, 20 for pupae and adults. We thus expect samples with fewer individuals to show greater biological variance and lower power than samples with more individuals.

Pupae and adults were produced by mated females and sexed by visual examination prior to extraction and expression profiling. Since sexing by visual examination is impossible before the pupal stage, male embryonic and larval samples were collected from virgin females, which produce only males. Female embryonic and larval samples were collected from mated females, which produced ~89.5% female offspring.

Expression values were measured via dual-channel whole-genome tiling path microarrays using custom NimbleGen high-density 2 (HD2) arrays [83], consisting of 8.4 million probes with a 50–60 bp length spanning the *Nasonia* genome at 33 bp intervals, as well as 27,000 Markov probes which are absent from the genome for noise detection (see below). Further details on animal breeding, RNA extraction and microarray processing are available in the supplementary materials of [46]. The microarray intensity files are available on NCBI with accession number GSE44701.

### Data pre-processing

Individual microarray probes were assigned to exons according to the latest release of the Official Nasonia Gene Set (OGS2.0) [58]. Expression for each exon was measured as the log2 ratio of the 99th quantile of the random Markov probes on their arrays. A sensible expression cut-off was set by examining the distribution of exon expression values across the whole experiment. Based on this assessment, all values below the 66th expression percentile were collapsed to zero to avoid spurious signal from random noise variation among non-expressed exons. We also retained only exons that showed expression above this signal threshold in at least two out of three replicates for at least one biological condition were retained for analysis.

Therefore, of the 126,213 annotated exons and 23,149 annotated genes from OGS2.0, only 67,409 exons and 14,151 genes were retained for investigating their transcriptional profiles after taking these quality assurance measures. Two more genes were filtered out as the result of quality control downstream in the pipeline; gene NASVI2EG009090 was removed during isoform switch detection (see below) since it showed low variation in expression across samples. Gene NASVI2EG021272 was removed before result interpretation because of its low annotation quality in OGS2.0. As such, the final dataset presented includes a total of 14,149 genes.

### Isoform switch detection

We applied the FESTA algorithm [84] to disentangle transcription and isoform switching signals. Briefly, FESTA allows detection of isoform switching based on experiment-specific exon expression data. FESTA disentangles signal from isoform switching (creating **‘splicing-nodes’**) from whole-gene transcription (**‘transcription-nodes’**) by first identifying constitutive exons that are present across all isoforms, then by measuring the relative frequency of facultative exons. We define facultative exons here as exons which are present in only some of the transcripts produced by each gene across the whole experiment. We choose to convert the expression values of splicing nodes to splicing ratios, which are normalized to the total expression value of the main gene within the same sample. Splicing ratios represent the proportion of all transcripts produced by a single gene which include the exons of interest. This allows splicing nodes to be represented as continuous proportions in a 0–1 range and removes the correlation between overall gene expression and expression of isoforms containing the exons of interest.

The final dataset contains a total of 36,505 nodes, 14,149 of which are transcription nodes and 22,356 of which are splicing nodes. Since each node is representative of a putative transcript, we refer to them using the term node and transcript interchangeably.

We subsequently collapsed all nodes with reciprocal correlation values greater than 95% into Constitutively Correlated Regulatory Events (CCREs) using the ‘collapseRows’ function from the WGCNA package implemented in R [36]. This step helped to reduce the dimensionality of our dataset by representing sets of nodes with almost identical expression patterns as single units. Each CCRE thus represents the expression pattern of a set of transcripts which behave identically to each other within our experiment. This approach is conceptually similar to that of Constitutively Co-expressed Links (CCELs) in [85]. We chose to represent each CCRE using expression scores of the node with the most correlation-based connections to other nodes in the same CCRE, because this value is the most representative of the average behavior of other CCRE members. In the special case were CCREs contained only two nodes, we chose the highest mean expression value. A total of 15,792 nodes was grouped into 3699 CCREs. As such, the final dataset features a total of 24,412 features of which 8720 (36%) are transcription nodes, 11,993 (49%) are splicing nodes and 3699 (15%) are CCREs. The complete annotation of all transcripts assigned to each CCRE is available in S3 File.

### Network construction

We constructed an undirected weighted interaction network of transcription-nodes and isoform-nodes applying the R package WGCNA [36] to the transcriptomic data of all whole-organism samples, both male and female. WGCNA infers between-node links based on power-transformed robust correlation scores. Since WGCNA does not require the input of pre-defined pathways or functional classes, it is ideally suited for the analysis of expression data from genomes with sparse functional gene annotations. WGCNA is also able to rapidly calculate large networks, which is a key feature for enabling permutation-based approaches to monitor differential correlations (see below).

Finally, we power-transformed pairwise correlation scores. This transformation increases the difference between weak and strong links in our network, with the effect of increasing overall specificity in detecting connected sub-networks [86]. Most natural network studies show a power-distribution of connectivity across nodes [87–89], with few highly connected nodes and many lowly connected ones, also called a scale-free degree distribution. Based on this “scale-free topology criterion” [86], we selected the lowest power that generated a scale-free correlation network (30).

### Network topology measurements

We measured two main network topology parameters for each node: connection densities and hub-scores.

**Connection density** is defined as the number of observed connections per node, normalized by the theoretical maximum possible number of connections. In an undirected weighted network, this parameter can be calculated with the formula $Kd_{i}=\sum k_{i}/\frac{N-1}{2}$ where $\sum k_{i}$ is the sum of the weights for all connections to node *i* and $\frac{N-1}{2}$ is the maximum number of links in an undirected network of size *N*. Connection density quantifies the relative importance of a node as a measure of its direct connections to other nodes within the same network (i.e. **Fig 8**, nodes A and C), and is useful to estimate the number of interactions among individual genes.

However, connection density does not account for the different regulatory potential of different connections. For instance, nodes A and B (**Fig 8**) have the same connection density. However, removing the connections from node A divides the network in two while removing the connections from node B causes no splitting in sub-networks, since its neighbors are already connected with each other.

To account for this topological property, we calculated **hub scores**, which measure the non-redundant connections provided by each node. We calculated hub scores using the formula $Hub_{i}=Kd_{i}\times \left(1-\frac{n_{i}}{\max\left(n_{i}\right)}\right)$ where *Kd*_*i*_ represents the connection density of node *i* and $\frac{n_{i}}{\max\left(n_{i}\right)}$ represents the clustering coefficient of node *i*, or the observed connectivity between nodes connected to node *i* divided by their maximum possible connectivity with each other. Nodes with high hub scores have a high number of connections to nodes that are otherwise unconnected among themselves and are likely to be involved in the coordination of multiple processes. Since we calculate hub scores by penalizing connection densities, a node’s hub scores cannot be higher than its connection density.

We use these two topological parameters to define three main classes of nodes in our network. **Marginal nodes** have low density and low hub scores (**Fig 8**, node C), and are unlikely to be involved in regulation. **Regulator nodes** have high density and high hub scores (**Fig 8**, node A), and are likely to be key regulators. Finally, **interactor nodes** have high density and low hub scores (**Fig 8**, node B), and form potentially functional stable associations with other nodes but are unlikely to be regulators.

To focus our investigation on the structure of local regulatory sub-networks across the sampled developmental stages, we calculated both scores by considering nodes within the same transcriptional cluster. We computed the within-cluster network statistics for each node using the ‘fundamental Network Concepts’ function from the WGCNA package, as well as weighted betweenness using the tnet package [90].

### Differential expression of nodes and clusters

We detected differential expression (DE) of individual nodes using generalized linear models (GLMs) as implemented in the LIMMA package [91]. We used the formula

Expression∼Stage+Stage:Sex

which accounts for stage-specific differences in gene expression via the factor *Stage* and considers sex only as a second-order interaction term with stage-specific expression changes (*Stage* : *Sex*).

To calculate cluster-level DE, we applied the same linear models to the first principal component (module eigengene) of each cluster. We performed multiple-hypothesis correction on both node and cluster-level DE results by converting the individual p-values to local False Discovery Rates (lFDR), which represent the individual probability of each hypothesis to be a false positive. We calculated lFDR using the R package fdrtool [92] on the p-values generated by LIMMA. All contrasts with a lFDR lower than 5% were considered significant. We classified each gene as sex-biased if at least one of its transcription- or splicing-nodes was differentially expressed (DE) between the sexes in at least one developmental stage. Full results for node-level analyses are included in S1 File.

### Testis and ovary bias detection

To detect which sex-biased isoforms are expressed in testes and ovaries, we contrasted the expression profile of male pupae and adult females with their respective gonads. We obtained the gene expression matrices of gonad samples by applying the same quality control procedure described above, and mapped their expression value to the nodes and CCREs we defined using the whole-animal samples to ensure we work with comparable datasets.

We detected differential expression using LIMMA GLMs [91] with the formula

Expression~sex−1*gonad

This formula thus calculates the baseline expression separately for each sex, contrasts them with their respective gonads, and finally checks whether the gonad-biased genes are more expressed in testes or ovaries. We selected for testis and ovary-enriched isoforms by applying the same false discovery rate (FDR) threshold as per the main analyses (5%) and by selecting only isoforms that are significant for both gonad bias and either testis or ovary bias. We classified each gene as ovary or testis-biased if at least one of its transcription or splicing-nodes were classified as testis or ovary biased.

We tested for enrichment of testis and ovary-biased isoforms in clusters by comparing the counts of testis and ovary-biased isoforms in clusters with at least five testis or ovary-biased isoforms with those in the rest of the transcriptome via Fisher’s exact test. We then corrected the p-values via FDR, and selected only clusters with an FDR smaller than 5%.

### Linkage clusters enriched by sex-biased loci

We next investigated whether sex-biased genes were enriching known linkage intervals, which would be indicative of sex-determining regions (SDRs) within the *Nasonia* genome. Since each node is DE tested independently at each stage, it is possible for a single gene to be both male and female-biased at different stages. Likewise, different transcription and splicing nodes from the same gene can show bias in either sex. Genes that fall in either category are unlikely to be subject to sex-specific selection, so we excluded them from linkage group enrichment analyses. We mapped all genes in our network to the linkage map published in [50], then tested each individual linkage group for enrichment in male or female-biased genes via one-tailed Fisher’s exact test, compared to the overall proportions of male and female-biased genes across all other linkage groups. This process generated two p-values per linkage group: one for female bias enrichment and one for male-bias enrichment. Finally, we applied FDR correction to the p-values using the package fdrtool, and reported all clusters with a lFDR score lower than 5%.

### Differential correlation analyses

In contrast to detecting differential expression of nodes based on significant changes in the relative abundance of transcripts, the detection of differential co-expression classifies groups of genes as biologically interesting based on a differential increase or decrease in their correlations across the conditions of interest (Fig 1). There are two distinct methods used to analyze differential co-expression: **‘untargeted’** methods identify changes in transcript-transcript interactions [34,85,93,94], and **‘targeted’** methods measure correlation changes in pre-defined groups of transcripts [35,95]. To allow direct comparisons between differential correlations and differential expression data, we developed a targeted method and applied it to the co-expression clusters found via network reconstruction.

The development of this strategy was necessary because most available methods are designed for two-sample tests, or to detect individual sample deviation from a pre-defined baseline [94,96,97], and are thus unable to account for multi-level and nested experimental designs. Moreover, untargeted methods are too computationally costly and underpowered for the comparisons of cluster-level DE.

Since our objective is to detect sex-specific gene co-regulation, we employed a sub-sampling strategy that removes all possible combinations of three samples within each stage, which equals the number of biological replicates for each stage by sex combination. This sub-sampling strategy retains a constant number of samples used for the generation of each sub-network, while systematically altering the proportion of samples from each sex at each developmental stage. For each sub-sampling iteration, the sub-networks were reconstructed using the R package WGCNA [36] using the same power transformation and node to cluster assignments as for the main network reconstruction (see **Network Construction** above). We then measured the within-cluster density of each cluster in every sub-sampled network. Since WGCNA-based cluster density is effectively a power-transformed measure of correlation between nodes in a cluster, we refer to its differential change as **‘differential correlation’** (DC).

Within-cluster density is a proportional measure that is distributed on a scale of 0 to 1, where 1 indicates that all possible connections between nodes are observed and where 0 indicates that no connections between nodes. The within-cluster density can therefore be analyzed using Generalized Linear Models (GLMs) with a gamma error distribution and logit link function. We fitted the following GLM to each cluster:

Density∼Stage+[Stage:Sex]+Network Density

This approach allowed the detection of stage-specific sex-bias in cluster density [*Stage*: *Sex*] while controlling for stage-specific and whole-network increases in connectivity. To validate that the observed density bias is non-random, we fitted the same GLM to 1000 datasets generated by randomly permuting sex-labels. The p-values for the [*Stage*: *Sex*] interactions were then calculated for each cluster from the GLMs of both the permuted and observed datasets. We then estimated the significance threshold for each case of differential correlation by calculating the lFDR of observed [*Stage*: *Sex*] p-values compared with the distribution of p-values generated by the randomly permuted dataset. Finally, we corrected for multiple-hypothesis testing by calculating, for each cluster, the [*Stage*: *Sex*] lFDR score against all other cluster lFDR scores. All [*Stage*: *Sex*] interactions with a lFDR score lower than 10% were deemed significant, leading to an expectation of making less than two false discoveries.

### Multivariate analysis of network parameters

Network and sub-network parameters display several non-trivial correlations [98,99]. Consequentially, we observe strong non-independence between our parameters of interest (Fig 5). We therefore employed a Principal Component Analysis (PCA) to detect the latent independent components that affect network parameters. The factors included in the PCA analysis are: cluster size (number of nodes), density, centralization, heterogeneity and median cluster coefficient as defined in [98], as well as cluster diameter (the longest among shortest paths within the network). We also included the relative proportion of splicing nodes and the relative proportion of nodes arising from duplicated genes as factors in the PCA analysis. Both of these proportions were normalized by their respective network-wide abundances before PCA. All variables were also centered and scaled before PCA.

Each of the principal components (PCs) extracted by PCA represents a single linear combination of the factors, which maximizes the degree of variance between clusters, and minimizes the reciprocal correlation with other PCs. We determined the biological significance of each PC by comparing the relative contribution of each parameter to their score (as estimated by parameter loadings). Since our objective is to determine whether any of the latent variables can discriminate between the different classes of sex-biased clusters, we used binomial GLMs including all PCs as predictors. We thus fit three separate model sets, using the following dependent variables: differentially expressed cluster, differentially correlated cluster, clusters with both differential expression and correlation. We then computed model sets containing all possible combinations of factors for each of the three main models and estimated each factor’s probability of being included in the best model of its set (their **relative importance**, or **RI**) using the small sample-size corrected version of the Akaike information criterion (AICc) based rankings as implemented by the R package MuMIn, [100]. Since only four clusters with differential correlations showed no differential expression in at least one stage, we only report results for the model set targeting differentially correlated clusters.

To detect whether any PC differs significantly between differentially expressed and differentially correlated clusters, we fitted a fourth binomial model set including only clusters with either differential expression or differential correlation, using differential correlation as a dependent variable and the eight PCs as its predictor.

### Phylostratigraphic analyses on network parameters

We used phylostratigraphic analysis to investigate the evolutionary age of genes within our networks [101] using annotations for *Nasonia* from [64] for hymenoptera and higher levels. Briefly, each gene is assigned the phylogenetic stratum of its deepest detectable homolog. Two genes are considered homologs if they have a blastp hit whose alignment covers at least 50% of the shorter protein with at least 30% positives (see [64]).

The hymenopteran species used are the ants (*Formicidae*) *Atta cephalotes*, *Acromyrmex echinatior*, *Camponotus floridanus*, *Harpegnathos saltator*, *Linepithema humile*, *Pogonomyrmex barbatus*, *Solenopsis invicta* and the bees (*Apidae*) *Apis florea*, *Apis mellifera*, *Bombus impatiens*, *Bombus terrestris*, *Megachile rotundata*. *Nasonia* is the only representative of the highly diverse and speciose Chalcidoidea superfamily [45,66]. Therefore, in this analysis, we assigned *Nasonia* genes without orthologs in the other hymenopterans the “chalcid” stratum. Higher level stratum assignments were based on eleven additional insects, two additional arthropods, and five additional metazoa species.

We performed a second identical set of analyses using the subset of genes included in the hymenopteran and chalcid strata, and adding more species to increase the phylogenetic resolution within hymenoptera. We used data from [65] for homology assignments to the chalcid species *Ceratosolen solmsi*, *Copidosoma floridanum*, and *Trichogramma pretiosum*, as well as the additional hymenopterans *Athalia rosae*, *Orussus abietinus*, and *Microplitis* demolitor. These additional data allowed separating genes that are found within chalcidoid wasps (matching with *Ceratosolen*, *Copidosoma* or *Trichogramma*), but not in the other Hymenoptera. Genes were assigned to the Chalcid stratum when an ortholog was found in one or more of the other chalcidoids, and to the *Nasonia* stratum when the *Nasonia* gene did not have any ortholog. Orthologs for this set were assigned using OrthoMCL with an e-value cutoff of 10e-10, percent match cutoff of 70 and inflation of 2.5 ([65], Supplementary Information, section S2.2).

Since few *Nasonia* genes from our prior stratum analysis matched to species within the Apocrita stratum (*Apis mellifera* and *Micropolitis demolitor*), we merged it with the closest older stratum (Hymenoptera). *Nasonia* genes which did not have homologs with any species within the set were assigned to the *Nasonia* stratum. All stratum assignments are available in S1 File.

We used GLMs to test for the impact of the inferred phylostratigraphic age on each node’s within-cluster connection density and hub scores by fitting the following models to each of the datasets:

ConnectionDensity∼ClusterSize+Stratum+DE+DC+[Stratum:DE]+[Stratum:DC] (1)

HubScore∼ClusterSize+Stratum+DE+DC+[Stratum:DE]+[Stratum:DC]. (2)

These models estimate the ability of taxonomic strata to predict connection density and hub scores both independently (term *Stratum*) and by interacting with two main sex-biased parameters (terms [*Stratum*: *DE*] and [*Stratum*: *DC*]), after controlling for variation in connection densities due to sex-bias parameters (terms *DE* and *DC*) and cluster size. Since connection densities and hub scores are expressed as 0–1 bound variables, we used a gamma error distribution and a logit link function. We subsequently fitted all possible nested models and produced model-averaged parameter estimates and RIs for each factors using AICc based rankings (as implemented in [100]). Results from the Metazoan to Hymenoptera stratum assignments are show in S4 File, and results from the Hymenoptera to *Nasonia* stratum assignments in S5 File.

### Gene ontology, and protein family enrichment analyses

For the Gene Ontology (GO) and PFAM (Protein Family database) enrichment tests, we used the interface provided by Wasp Atlas, which returns FDR-corrected q-values for over-representation of GO and PFAM categories in the gene-set of interest by using one-tailed FDR corrected hypergeometric over representation tests [56]. We choose a significance threshold of q < 0.01. The input files used for enrichment testing were either lists of genes (for linkage group enrichment) or transcription nodes (for transcriptional cluster enrichment).

## Supporting information

S1 File Complete annotation of *Nasonia* transcripts described in the experiment. (ZIP)

S2 File Complete annotation of *Nasonia* transcriptional clusters. (ZIP)

S3 File Annotation of transcripts comprising constitutively co-expressed regulatory events. (ZIP)

S4 File Coefficients and importance of phylostratigraphic age on network parameters, using strata from Metazoa to Chalcid. (CSV)

S5 File Coefficients and importance of phylostratigraphic age on network parameters, using strata from Hymenoptera to *Nasonia*. (CSV)
